# The Islanding effect - a special method of percutaneous peritumor ethanol injection for hepatocellular carcinoma: 15-year follow-up outcome

**DOI:** 10.1097/MD.0000000000024365

**Published:** 2021-01-22

**Authors:** Ze-Wu Meng, Xin-Ran Cai, Chang-Zhao Lin, Yan-Ling Chen, Song Liu

**Affiliations:** Fujian Medical University Union Hospital, Department of Hepatobiliary Surgery and Fujian Institute of Hepatobiliary Surgery, Fujian Medical University Cancer Center, Fuzhou, Fujian, People's Republic of China.

**Keywords:** hepatocellular carcinoma, percutaneous peritumor ethanol injection, prognostic factor, recurrence, survival

## Abstract

Percutaneous ethanol injection is a well-known ablation therapy for hepatocellular carcinoma and is well-tolerated, inexpensive, and effective with few adverse events. In this study, another type of ethanol injection was introduced in the present study.

Sixty two patients with hepatocellular carcinoma received 133 percutaneous peritumor ethanol injection treatments and the 15-year follow-up outcomes were analyzed through a collected database.

The technical efficiency was 89.5% (119/133 treatments) after the first percutaneous peritumor ethanol injection procedure. However, after the second repeated percutaneous peritumor ethanol injection procedure, technical efficiency increased to 98.5% (131/133 treatments). The 1 year, 3 years, 5 years, 10 years, and 15 years rates of tumor recurrence were 12.9%, 50.0%, 59.7%, 74.2%, and 74.2%, respectively. Multivariate analysis demonstrated that diabetes, Child–Pugh class B, and tumor size greater than 2 cm were significantly related to tumor recurrence. The 1 year, 3 years, 5 years, 10 years, and 15 years rates of overall survival were 98.4%, 83.6%, 61.3%, 19.4%, and 0%, respectively. Multivariate analysis demonstrated that Child–Pugh class B, tumor size greater than 2 cm, and multiple tumors were significantly related to overall survival.

Compared with other ablation methods (including peritumor ethanol injection), percutaneous peritumor ethanol injection can avoid tumor ruptures, reduce tumor proliferation and metastasis, and is suitable for the treatment of small tumors. In addition, when combined with other treatment methods, percutaneous peritumor ethanol injection can form a tumor metastatic isolation zone in advance and improve the comprehensive treatment effect.

## 1 Introduction

Hepatocellular carcinoma (HCC) is most common in Asian countries, mainly because of the high incidence of viral hepatitis.^[1]^ With the control of viral hepatitis B, the incidence of HCC in China has decreased, but the mortality rate is still high, and the prognosis is poor.^[2]^ Liver resection is still one of the most effective treatment methods. For some unresectable patients, the treatments include percutaneous ethanol injection (PEI),^[3]^ radiofrequency ablation (RFA),^[4]^ cryoablation therapy,^[5]^ percutaneous acetic acid injection,^[6]^ transcatheter arterial embolization, and microwave coagulation therapy.^[7]^ Among all these therapies, PEI therapy is still widely applied because it is well-tolerated, inexpensive, and effective with few adverse events.^[3]^

Although PEI had been introduced into clinical practice as early as 1980,^[8,9]^ the injection mode, such as injecting ethanol into several sites in and around the tumor and repeating until ethanol appeared to have been injected throughout the tumor, has not changed.^[3,10,11]^ In this study, a special ethanol injection method for peritumor tissue, guided by computed tomography (CT) that does not touch and destroy the tumor capsule. After this percutaneous peritumor ethanol injection (PPEI) method, the tumor presents an island appearance in contrast to the background of the peritumor ethanol in the CT scan. Therefore, it is also called the “islanding effect”. Compared with other ablation therapies, this method does not touch and destroy the tumor capsule and achieves similar radical treatment by inactivating the surrounding tissues of the tumor. Therefore, we summarize a 15-year consecutive case series study of this injection method to illustrate the operation, advantages, disadvantages, and precautions, which may then allow PPEI therapy to be more helpful in the comprehensive treatment of HCC.

## 2 Patients and methods

### 2.1 Patients

The retrospective data of patients who underwent the PPEI for the first diagnosed HCC were enrolled from January 2002 to January 2018 at Fujian Medical University Union Hospital (Fujian, China). Inclusion criteria:

1. All patients with HCC were diagnosed by at least 2 different imaging examinations;
2. only PPEI, not PEI, therapy was given throughout the treatment;
3. tumor diameter <5 cm and tumor number ≤2;
4. no previous or simultaneous malignancies except HCC;
5. no extrahepatic metastasis or vascular invasion.

Exclusion criteria:

1. Child–Pugh class C;
2. Intrahepatic cholangiocarcinoma, mixed type of hepatocarcinoma, metastatic liver tumors, and tumors of uncertain origin;
3. The puncture needle punctured the tumor during ethanol injection treatment, causing ethanol infiltration and tumor dissociation;
4. Follow-up data were incomplete.

Finally, 62 patients were enrolled in the study, and the baseline data are summarized in Table 1.

**Table 1:** Baseline characteristics of 62 HCC patients undergoing PPEI therapy.

Characteristic	
Age (years)	55.0 ± 11.2
Males, n (%)	56 (90.3)
HBs-Ag positive, n (%)	54 (87.1)
Alcohol consumption >80 g/day, n (%)	4 (6.5)
Diabetes, n (%)	10 (16.1)
Family history of cancer, n (%)	11 (17.7)
Prothrombin time activity (%)	75.1 ± 12.8
Platelet count (×10^4^/mm^3^)	11.2 ± 6.3
Total bilirubin (umol/L)	27.0 ± 18.5
AST (IU/L)	43.8 ± 37.9
ALT (IU/L)	46.9 ± 34.0
Albumin (g/L)	38.7 ± 5.9
Child–Pugh class, n (%)	
A	55 (88.7)
B	7 (11.3)
Serum AFP (ng/ml), n (%)	
≤100	37 (59.7)
>100	25 (40.3)
Tumor size (cm)	1.9 ± 0.8
Tumor number	1.1 ± 0.3

The following variables were extracted: age (≤60 years, >60 years), sex, hepatitis B status, alcohol consumption (≤80 g/day, >80 g/day), diabetes, family history of cancer,^[12]^ prothrombin time activity (<75%, ≥75%), platelet count (<105/mm^3^, ≥105/mm^3^), total bilirubin (≤21 μmol/L, >21 μmol/L), AST (≤40 IU/L, >40 IU/L), ALT (≤40 IU/L, >40 IU/L), albumin (<35 g/L, ≥35 g/L), Child–Pugh class (A, B), serum a-fetoprotein (AFP) (≤100 ng/ml, >100 ng/ml), tumor size (≤2 cm, >2 cm), and tumor number (solitary, multiple). The tumor size was measured as the largest diameter of multiple tumors.

This study was approved by the Ethics Committee of the Medical Faculty of Fujian Medical University in China (no. 2018KY066). Informed consent was obtained from each patient before PPEI.

### 2.2 Treatment methods

**Figure 1:**
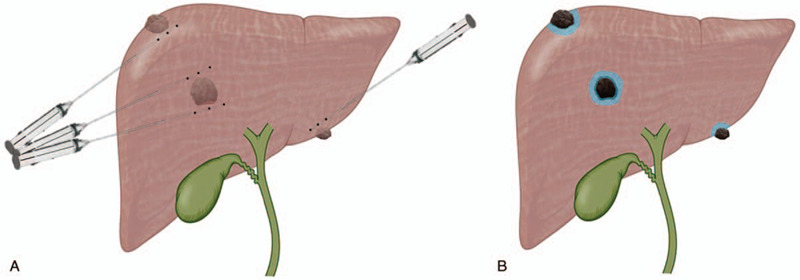
The selection of the puncture route and alcohol injection site in PPEI therapy (A). A brief diagram of tumors after PPEI therapy: tumor vascular embolization, tumor atrophy and necrosis (B). PPEI = percutaneous peritumor ethanol injection.

Preoperative CT and magnetic resonance (MR) imaging was performed to identify the tumors and determine the puncture route. The procedure was performed by an experienced surgeon who was in charge of this operation in Fujian Medical University Union Hospital. All procedures were performed by CT guidance. The selection of the puncture route and ethanol injection site is shown in Figure 1. The volume of ethanol injected and the number of ethanol injection sites depended on the peritumor tissue boundary it penetrated, and the volume generally did not exceed 20 ml in 1 site. When ethanol is injected around the tumor, it usually spreads along the gap between the tumor and the surrounding normal tissue and solidifies the surrounding tissue to form the outermost barrier. Then, the later ethanol injection accumulates between the barrier and the tumor, without loss, to form a partial encirclement around the tumor. After injecting ethanol at multiple sites, a complete encirclement of the tumor is gradually formed. The PPEI procedure is completed when the low-density focus around the entire tumor (upper, lower, left, right, anterior, posterior) in the CT scan reaches at least 1 cm.

The volume of injected ethanol depends on the infiltrative edge of peritumor tissue reaching at least 1 cm during the operation. The following numerical expression can also be used for reference, V = (4/3) π [(*r* + 1)^3^ − (*r*)^3^], where V (milliliters) is the volume of ethanol and *r* (centimeters) is the radius of the tumor; 1 is the infiltrative safety margin of peritumor tissue. Then, after one month, an enhanced MR scan was performed to evaluate tumor activity. Complete ablation was defined as the entire tumor being nonenhanced in the enhanced MR scan. When enhanced portions of the tumor were suspected, repeated procedures were performed for the enhanced portions. Treatment usually continued until an enhanced MR scan revealed no enhancement of the entire tumor.

### 2.3 Follow-up

The definition of the follow-up period was from the date of PPEI to death or until January 2018. MR, ultrasound and serum AFP were performed every 6 months. If the tumor recurrence met the inclusion criteria, PPEI therapy was still applied. However, if PPEI therapy was not suitable for the treatment of multiple recurrences, chemoembolization was generally adopted. The disease-free survival (DFS) was calculated from the date of the first PPEI to the date of tumor recurrence due to HCC. Patients who had no tumor recurrence were censored on the date of last contact for DFS outcomes. Overall survival (OS) was calculated from the date of the first PPEI to the date of death due to any cause. Patients who were alive were censored on the date of last contact for OS outcomes.

### 2.4 Statistical analysis

A “procedure” was defined as a single ethanol injection into the peritumor, and a “treatment” was defined as the total number of ethanol injections performed for the complete necrosis of the tumor. “Technical efficiency” was defined as the percentage of complete tumor necrosis shown on the MR scan after every procedure. If the tumor was completely nonenhanced in the final MR scans, we considered that the tumor was completely necrotic. The definition of complications was based on the Guidelines of the Society for Interventional Radiology.^[13]^

The patients’ clinical data were analyzed using SPSS 20.0 software. The data of quantitative variables were expressed as the mean ± standard deviation (SD), and the data of qualitative variables were expressed as absolute frequency. Survival curves of DFS and OS were drawn. The Cox regression model was used to evaluate the risk of HCC after PPEI therapy. All significance tests were two-tailed, and a *P* value ≤.05 was considered statistically significant.

## 3 Results

### 3.1 Peritumor PPEI effect

A total of 62 HCC patients underwent PPEI therapy, and some of them received iterative PPEI therapy for recurrence. We performed 150 ethanol injection procedures and 133 ethanol injection treatments. There were 28 patients who underwent PPEI treatment once, 15 patients twice, 8 patients 3 times, 5 patients 4 times, 1 patient 5 times, 2 patients 6 times, and 2 patients 7 times. One patient underwent 3 ethanol injection procedures, but the tumor was still active and was then treated with chemoembolization; this patient was only counted as an ethanol injection procedure, not as an ethanol injection treatment. The ethanol injection volume was 33.9 ± 14.0 ml per procedure. The technical efficiency was 89.5% (119/133 treatments) after the first PPEI procedure. However, after the second repeated PPEI procedure, technical efficiency increased to 98.5% (131/133 treatments).

### 3.2 Disease-free survival and overall survival

**Figure 2:**
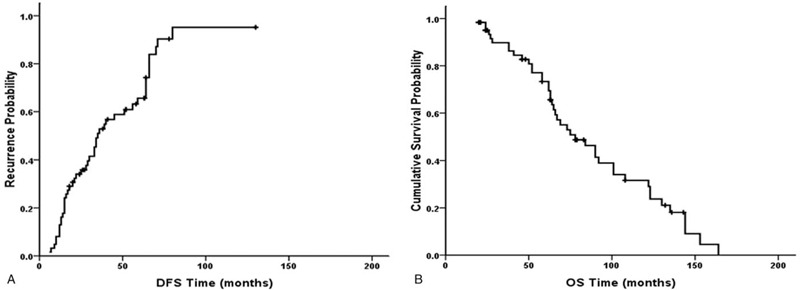
Survival curves of DFS (A) and OS (B) for HCC after PPEI therapy. DFS = disease-free survival, HCC = hepatocellular carcinoma, OS = overall survival, PPEI = percutaneous peritumor ethanol injection .

Tumor recurrence developed in 46 patients, intrahepatic recurrence developed in 43 patients and extrahepatic recurrence developed in 3 patients. For the first recurrence in these patients, 33 (71.7%) underwent repeated PPEI therapy, 8 (17.4%) underwent chemoembolization, and 5 (10.9%) underwent supportive treatment. The 1-, 3-, 5-, 10-, and 15-year rates of tumor recurrence were 0.81%, 52.8%, 65.6%, 95.2%, and 95.2%, respectively (Fig. 2A). Multivariate analysis demonstrated that diabetes, Child–Pugh class B, and tumor size greater than 2 cm were significantly related to tumor recurrence (Table 2).

**Table 2:** Multivariate COX analysis of disease-free survival and overall survival of HCC patients undergoing PPEI therapy.

	Disease-free survival			Overall survival		
Characteristic	HR	95% CI	*P*^∗^	HR	95% CI	*P*^∗^
Age (> 60)	0.846	0.364–1.965	.697	1.053	0.396–2.799	.917
Sex (Female)	2.366	0.760–7.369	.137	1.534	0.453–5.201	.492
HBs-Ag positive	0.902	0.315–2.586	.848	1.668	0.546–5.095	.369
Alcohol >80 g/day	1.036	0.152–7.080	.971	1.740	0.259–11.692	.569
Diabetes	3.305	1.185–9.215	**.022**	2.391	0.657–8.710	.186
Family history of cancer	0.965	0.356–2.619	.944	1.586	0.573–4.383	.374
Prothrombin time >115%	0.987	0.410–2.373	.976	1.654	0.594–4.610	.336
Platelet count < 10^5^/mm^3^	0.775	0.304–1.976	.593	1.038	0.384–2.802	.942
Total bilirubin >21 umol/L	1.366	0.642–2.908	.418	1.650	0.659–4.133	.285
AST > 40 IU/L	1.531	0.453–5.171	.493	1.140	0.343–3.790	.831
ALT > 40 IU/L	1.411	0.407–4.894	.588	1.206	0.347–4.187	.769
Albumin < 35g/L	0.697	0.262–1.853	.469	2.356	0.886–6.263	.086
Child–Pugh class B	4.134	1.003–17.040	**.050**	14.638	2.618–81.843	**.002**
Serum AFP > 100 ng/ml	0.919	0.382–2.214	.851	1.991	0.843–4.699	.116
Tumor size > 2cm	2.504	1.063–5.899	**.036**	3.339	1.250–8.920	**.016**
Multiple tumors	11.009	0.977–124.017	.052	27.343	1.281–583.625	**.034**

Until January 2018, 19 (30.6%) patients remained alive, and 43 (69.4%) had died. The median follow-up time was 87.8 months. The causes of death were 29 (67.4%) cases of HCC, 7 (16.3%) cases of liver failure, 4 (9.3%) cases of upper gastrointestinal bleeding, and 3 (7.0%) cases of unrelated liver diseases. The 1-, 3-, 5-, 10-, and 15-year overall survival rates of all 62 patients were 98.4%, 83.6%, 61.3%, 19.4%, and 0%, respectively (Fig. 2B). Multivariate analysis demonstrated that Child–Pugh class B, tumor size more than 2 cm, and multiple tumors were significantly related to overall survival (Table 2).

### 3.3 Complications

In the 150 PPEI procedures, sustained fever higher than 37.5°C for more than 3 days occurred in 48 procedures. A serious complication, massive hepatic infarction, occurred in 1 PPEI procedure. It presented irregular massive hepatic infarction in the right posterior lobe of the liver due to the embolism of the right posterior portal vein. After conservative treatment for liver protection, liver function was recovered. Other serious complications, such as neoplastic seeding, hemoperitoneum, hemobilia, and liver abscess, did not occur.

## 4 Discussion

As stated in the introduction, the classical therapy of PEI is ethanol injection throughout the tumor. PPEI, in comparison, has advantages and disadvantages but may provide an available method of ethanol injection for some cases.

When patients chose to be treated with ethanol injection, we first considered PPEI. If the needle punctured the tumor during the PPEI procedure, causing ethanol infiltration and tumor dissociation, then PEI was chosen, and the patient was not enrolled. Additionally, if the tumor was in a position in which the ethanol injection could not form a perfect surrounding “islanding effect”, we considered injecting the peritumor first to form a partial encirclement around the tumor and then puncturing the rear of the tumor to form the rest of the encirclement. After the whole encirclement was formed, ethanol was injected into the tumor to ablate the tumor. We considered treating the peritumor tissue first and the cancerous tissue last to reduce the possibility of tumor spread and metastasis, but this was only a conjecture that needs further comparison and verification. We only selected patients who underwent PPEI for the first time, instead of PEI; thus, the number of cases was relatively small. A total of 62 patients were enrolled in the study (Table 1). The study showed that PPEI could also achieve long-term survival (Fig. 2).

**Figure 3:**
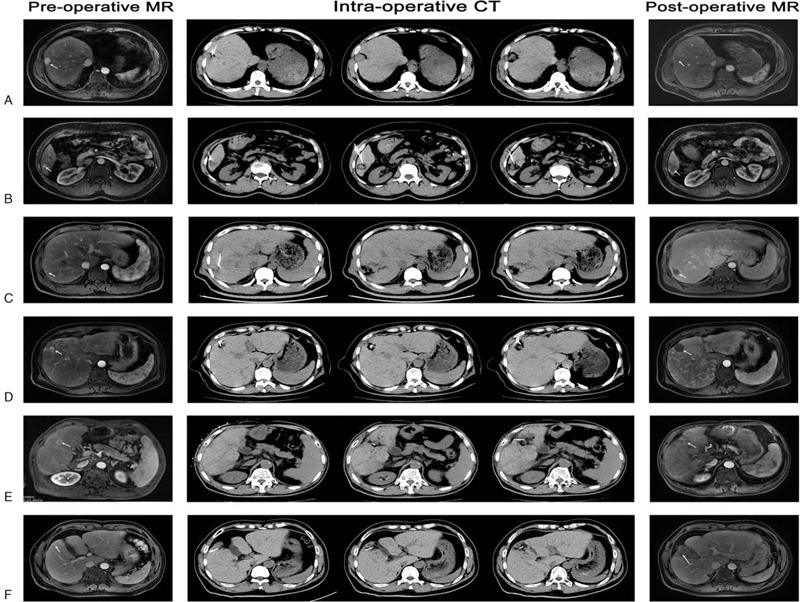
The preoperative MR (arterial phase), intraoperative CT (plane scanning) and postoperative month MR (arterial phase) of 6 HCC patients undergoing PPEI therapy. A, male, 38 years old, hepatocellular carcinoma in segment 8 of liver, tumor size: 3.1 × 2.6 × 2.3 cm, volume of ethanol injection: 55 ml. B, male, 52 years old, hepatocellular carcinoma in segment 6 of liver, tumor size: 1.6 × 1.4 × 1.2 cm, volume of ethanol injection: 29 ml. C, male, 40 years old, hepatocellular carcinoma in segment 7 of liver, tumor size: 0.9 × 0.8 × 0.7 cm, volume of ethanol injection: 20 ml. D, male, 63 years old, hepatocellular carcinoma in segment 5 of liver, tumor size: 1.7 × 1.6 × 1.4 cm, volume of ethanol injection: 32 ml. E, male, 61 years old, hepatocellular carcinoma in segment 6 of liver, tumor size: 2.4 × 2.1 × 1.7 cm, volume of ethanol injection: 43 ml. F, female, 50 years old, hepatocellular carcinoma in segment 5 of liver, tumor size: 2.1 × 1.4 × 1.2 cm, volume of ethanol injection: 33 ml. CT = computed tomography, HCC = hepatocellular carcinoma, MR = magnetic resonance, PPEI = percutaneous peritumor ethanol injection.

In these patients, 150 PPEI procedures and 133 PPEI treatments were obtained. The technical efficiency was 89.5% (119/133 treatments) after the first PPEI procedure. However, after the second repeated PPEI procedure, technical efficiency increased to 98.5% (131/133 treatments). The proportion in this study was comparable to studies of other ablation therapies (92%–98.2%),^[3,14,15]^ which shows that the PPEI method is feasible. The 5- and 10-year rates of tumor recurrence after PPEI therapy were 65.6% and 95.2%, respectively (Fig. 2A). In patients treated by other ablation therapies, the 5- and 10-year rates of tumor recurrence were 59.0% to 96.0% and 91.9%, respectively. The 5- and 10-year overall survival rates after PPEI therapy were 61.3% and 19.4%, respectively (Fig. 2B). In patients treated by other ablation therapies, the 5- and 10-year overall survival rates were 30.0% to 61.0% and 17.9%, respectively. Although this was an uncontrolled observational study, tumor recurrence, and survival after PPEI therapy were similar to those after other ablation therapies.^[3,10,16–20]^ Additionally, multivariate analysis demonstrated that diabetes, Child–Pugh class B, and tumor size more than 2 cm were significantly related to tumor recurrence, and Child–Pugh class B, tumor size more than 2 cm, and multiple tumors were significantly related to overall survival (Table 2). The results were similar to those from studies of other ablation therapies.^[3,10,16,17,21–24]^ The preoperative MR (arterial phase), intraoperative CT (plane scanning) and postoperative month MR (arterial phase) of 6 HCC patients undergoing PPEI therapy are shown in Figure 3.

Compared to other ablation therapies, PPEI has advantages. First, for some exogenous and subcapsular HCC, PEI, and RFA may cause tumor ruptures, leading to intraperitoneal spread or perihepatic organic injury.^[10,25]^ The PPEI-targeted injection was peritumor tissue, which did not destroy the tumor and liver capsule and did not lead to the possibility of intraperitoneal spread (Fig. 3A and 3B) or perihepatic organic injury (Fig. 3F). Second, if the tumor size is less than 1 cm, it is not easy to find the exact location for ablation therapy. However, after covering the target area with ethanol, the general outline of the tumor will present in the low-density area (Fig. 3C), and it can be treated after the peritumor ethanol injection. Third, due to the limitation of the tumor capsule, the injected ethanol is mainly distributed in the tumor and is not easily spread to the peritumor tissue, so it has little impact on the peritumor tissue. The PPEI mainly targeted the peritumor tissue and peritumor micro tumor embolus, and the infiltrative safety margin of peritumor tissue (1 cm) was larger than the original PEI (0.5 cm),^[3,11,26]^ similar to the surgical safe limit for resection. Peritumoral microvascular invasion is considered to be the key of tumor recurrence and metastasis.^[19]^ However, PPEI makes the tumor more prone to ischemic necrosis and destroys the tissue where the peritumoral microvascular invades and metastases (Fig. 3D and 3E). After PPEI therapy, the 5-year rate of tumor recurrence and overall survival were 65.6% and 61.3%, respectively. Compare with previous studies, the 5-year rate of tumor recurrence and overall survival were 63.8% and 71.1% in the hepatic resection group, 71.7% and 61.1% in the RFA group, and 76.9% and 56.3% in the PEI group, respectively.^[27]^ Therefore, in terms of the 5-year rate of tumor recurrence after PPEI, it is superior to PEI and RFA. In addition, the PPEI does not touch and puncture the tumor to prevent spreading due to the high injection pressure in the tumor, which increases the possibility of distant metastasis. Fourth, repeated ablation therapy increases the chances of neoplastic seeding along the needle tract and may induce serious complications, such as gastrointestinal bleeding and liver failure.^[28]^ PPEI guided by CT does not require repeated injections over several days,^[3]^ thus reducing the length of the hospital stay and the pain of patients. Moreover, the tumor was not touched, so there was no risk of needle metastasis if multiple punctures were involved in a single injection procedure. Even though the tumor was still active after the initial injection, the peripheral peritumor tissue had formed a necrotic surrounding zone, and the tumor could not metastasize outward during the observation process 1 month after the therapy. Moreover, the new computational formula, V = (4/3) π [(*r* + 1)^3^ − (*r*)^3^], uses less ethanol and causes less liver damage than the original formula, V = (4/3) π (*r* + 0.5)^3^. The ethanol injection volume of PPEI (33.9 ± 14.0 ml) in this study was less than that of the original PEI (40.9 ± 16.3 ml) in another study.^[3]^ The PPEI procedure requires fewer injections and less ethanol volume than the other ablation therapies, so there are fewer complications. A serious complication, massive hepatic infarction, occurred in only 1 PPEI procedure. Other serious complications, such as neoplastic seeding, liver abscess, hemoperitoneum, and hemobilia,^[3,10,16,25,29]^ did not occur.

Compared to other ablation therapies, PPEI also has disadvantages. First, the successful use of PPEI is constrained by many factors, such as tumor location, depth of ethanol penetration, experience of the operator, and tumor size. Second, if the tumor is still active after 3 PPEI procedures, PPEI therapy is not recommended again. We considered that ethanol could not penetrate into the thick wall of the artery around the tumor to block its blood supply. At this point, combined with other treatments, such as transcatheter arterial embolization and RFA, good results could be achieved because the surrounding necrotic boundary has been established and the tumor cannot easily spread. Third, the injection procedure must be guided by CT. If the tumor is large, or if the procedure is long, which may cause the patient to bear more CT radiation, multipoint injection should be selected. Fourth, peritumoral injection is likely to lead to ethanol embolization directly into the main branch of the portal vein and cause massive hepatic necrosis. However, skillful technique can effectively reduce the incidence of this complication. Therefore, when the needle tip reaches the injection site, we first inject 3 to 5 ml ethanol to determine whether there is a branch of the main blood vessels. If ethanol was injected into the branch of the main blood vessel, intravascular thrombosis would form instantly. Then, we continued to inject the needle a little deeper, deviating from the original position, continuing to inject the ethanol so it would not flow into the branch of the main blood vessels. Fifth, multivariate analysis demonstrated that tumor size greater than 2 cm was significantly related to tumor recurrence and overall survival (Table 2). This indicates that PPEI therapy has a good effect on tumors smaller than 2 cm, similar to PEI therapy.^[10]^

PPEI has precautions. First, the slope direction of the needle tip needs to be adjusted to change the ethanol trend. Generally, ethanol will spread along the slope of the needle tip. Therefore, the slope of the needle tip should be inclined toward the tumor during the injection. Second, if the HCC has an equal density in the plain CT scan, the location can be determined by various hepatic duct and vessel structures, perihepatic markers, small intrahepatic cysts and other indicators. After covering the target area with ethanol, the island structure will present in the low-density area. That is, the general outline of the tumor. Third, PPEI requires more experience for the operator. Because breathing movements may easily lead to deviation in the position of the injected location, injections at more sites may be required to obtain a good surrounding effect. The experienced operator can achieve high efficiency with 1 puncture.

There are several limitations to this study. First, further matched case-control studies, including multiple centers and more patients, are needed to confirm these results and better understand the indications for this technique. Second, this study was limited to patients with relatively good liver function because patients with Child-Pugh class C were excluded. Third, the study population cannot be clearly defined. This study lasted for fifteen years. Along with the introduction of the other ablations, the indication criteria of ethanol injection changed over time.

In conclusion, compared with other ablation methods, PPEI can avoid tumor ruptures, reduce tumor proliferation and metastasis, and is suitable for the treatment of small tumors. When combined with other treatment methods, it can also form a tumor metastasis isolation zone in advance to prevent tumor metastasis and improve the therapeutic effect. The efficacy of PPEI needs to be further validated, but it provides a new modality that may provide better efficacy when treated alone or in combination.

## Author contributions

**Accountable for all aspects of the work:** All authors.

**Collection and assembly of data:** All authors.

**Conception and design:** Ze-Wu Meng, Song Liu.

**Data analysis and interpretation:** Ze-Wu Meng, Chang-Zhao Lin, Xin-Ran Cai.

**Final approval of manuscript:** All authors.

**Financial support:** Ze-Wu Meng, Yan-Ling Chen.

**Manuscript writing:** Ze-Wu Meng, Xin-Ran Cai.

**Conceptualization:** Ze-Wu Meng, Yan-Ling Chen, Xin-Ran Cai, Song Liu.

**Data curation:** Ze-Wu Meng, Yan-Ling Chen, Chang-Zhao Lin, Song Liu.

**Formal analysis:** Ze-Wu Meng, Xin-Ran Cai.

**Funding acquisition:** Ze-Wu Meng, Yan-Ling Chen.

**Investigation:** Ze-Wu Meng, Yan-Ling Chen, Xin-Ran Cai, Chang-Zhao Lin, Song Liu.

**Methodology:** Ze-Wu Meng, Xin-Ran Cai, Chang-Zhao Lin, Song Liu.

**Project administration:** Ze-Wu Meng, Yan-Ling Chen, Xin-Ran Cai.

**Resources:** Ze-Wu Meng, Yan-Ling Chen, Chang-Zhao Lin, Song Liu.

**Supervision:** Yan-Ling Chen, Song Liu.

**Validation:** Ze-Wu Meng, Yan-Ling Chen, Xin-Ran Cai, Song Liu.

**Visualization:** Yan-Ling Chen, Song Liu.

**Writing – original draft:** Ze-Wu Meng, Yan-Ling Chen, Xin-Ran Cai.

**Writing – review & editing:** Ze-Wu Meng, Yan-Ling Chen, Song Liu.
